# Biomarkers and Nutrients in Musculoskeletal Disorders

**DOI:** 10.3390/nu13020283

**Published:** 2021-01-20

**Authors:** César Calvo-Lobo, Ricardo Becerro-de-Bengoa-Vallejo, Marta Elena Losa-Iglesias, David Rodríguez-Sanz, Daniel López-López, Marta San-Antolín

**Affiliations:** 1 Facultad de Enfermería, Fisioterapia y Podología, Universidad Complutense de Madrid, 28040 Madrid, Spain; cescalvo@ucm.es (C.C.-L.); ribebeva@ucm.es (R.B.-d.-B.-V.); 2 Faculty of Health Sciences, Universidad Rey Juan Carlos, 28922 Alcorcón, Spain; marta.losa@urjc.es; 3 Research, Health and Podiatry Group, Department of Health Sciences, Faculty of Nursing and Podiatry, Universidade da Coruña, 150403 Ferrol, Spain; daniel.lopez.lopez@udc.es; 4 Department of Psychology, Universidad Europea de Madrid, 28670 Madrid, Spain; marta.sanantolin@universidadeuropea.es

Worldwide, the burden of musculoskeletal disorders is increasing with great variations between-countries, which makes it difficult for policymakers to provide resources and adequate interventions in order to provide for their appropriate management [1]. Thus, musculoskeletal disorders remain a public health problem and their incidence and trend is increasing for some specific conditions [2].

Indeed, nutrients and biological biomarkers play a key role in the prognosis, diagnosis and health status of patients suffering from musculoskeletal conditions, being the main indicators for understanding biological processes as well as tailoring therapeutic interventions and nutritional programs in patients with musculoskeletal disorders [3].

Thus, the purpose of this Special Issue was to provide an update on the state of the art, through current reviews as well as new insights and interventions, about the main role of nutrients and biomarkers in patients who suffer from musculoskeletal conditions from a multidisciplinary point of view. According to the aim of this Special Issue, a total of six papers summarized in Table 1 and Table 2 were published between May and December, 2020, in Nutrients. 

Among these publications, three papers were reviews providing an update of various topics including the current metabolic impact of COVID-19 pandemic confinement secondary to diet modifications and the reduction of physical activity [4]; the influence of nutrients for osteoporosis prevention in a specific population of patients suffering from inflammatory bowel disease [5]; and the relationship of nutrient and metabolic status with spinal muscular atrophy [6]. 

In addition, three papers were original research studies supporting key information from a multidisciplinary approach about the effects of curcumin and resveratrol on satellite cells as well as biomarkers for muscle regeneration process [7]; the fat rich diet effect on acetylcholine spontaneous release produced in mice neuromuscular junctions revealing key aspects for the relationship myofascial pain syndrome and nutrients or biomarkers [8]; and the effects of supplementation with vitamin D and L-cysteine on the biomarkers of musculoskeletal conditions in high-fat-diet–fed mice with vitamin D deficiency [9].

The main characteristics of the reviews provided in this Special Issue are presented in Table 1, updating the state of the art of the available scientific evidence on nutrients and biomarkers in musculoskeletal conditions about trending topics such as the effects of the COVID-19 pandemic, the prevention of osteoporosis related to inflammatory bowel disease, as well as the influence of nutrients and biomarkers in spinal muscular atrophy [4,5,6].

Firstly, Martínez-Ferran et al. [4] provided an interesting review which detailed the key metabolic consequences of the COVID-19 confinement, such as increased insulin resistance, body and abdominal fat, as well as inflammatory cytokines, which are linked to metabolic syndrome development and the appearance of musculoskeletal disorders, among others. The authors encouraged researchers to explore positive energy balance as a plausible mechanism for these nutritional and musculoskeletal disorders, and suggested that the restriction of calorie intake could prevent the COVID-19 confinement impact associated with physical inactivity. 

Secondly, Ratajczak et al. [5] reviewed the influence of nutrients in preventing osteoporosis and low bone mineral density in patients who suffered from inflammatory bowel disease, i.e. ulcerative colitis and Crohn’s disease. Vitamin D and calcium were the most commonly studied nutrients for bone mineral density. In addition, vitamins such as A, B_12_, C and K, calcium, folic acid, magnesium, phosphorus, zinc, sodium, selenium and copper were implicated in bone mass formation. These patients seemed to commonly consume inadequate amounts of these vitamins and minerals, impairing absorption and disturbing nutritional status with an increase in the risk of osteoporosis. Therefore, the authors also encouraged researchers to develop nutritional guidelines for patients suffering from inflammatory bowel diseases to prevent osteoporosis.

Lastly, the review carried out by Li et al. [6] highlighted the relationship between nutrient and metabolic status and neurodegenerative diseases. Concretely, various fatty acids’ metabolic alterations and glucose tolerance impairment were linked to spinal muscular atrophy. Thus, nutritional support as well as monitoring of biomarkers and nutrients may play a key role in patients who suffer from spinal muscular atrophy. Furthermore, metabolomics may provide a promising support as therapeutic targets or specific biomarkers for metabolic alterations and for the quantification of specific metabolites in patients who suffer from this neurodegenerative disease.

The principal characteristics of the original research reports published in this Special Issue are presented in Table 2, providing new insights and interventions about the key role of nutrients and biomarkers in patients who suffer from musculoskeletal conditions, dealing with specific main topics such as the importance of satellite cells in the process of muscle regeneration, the release of acetylcholine in the neuromuscular junction for the myofascial pain syndrome development, as well as nutritional supplementation for musculoskeletal disorders [7,8,9].

First, Mañas-García et al. [7] carried out an excellent experimental laboratory study detailing the curcumin and resveratrol effects on satellite cells by analyzing muscle regeneration biomarkers. Resveratrol and curcumin supplementation in the immobilized muscles of mice elicited an increase in muscle satellite cells. Curcumin treatment for reloaded muscles improved the cross-sectional area of hybrid muscle fibers and sirtuin-1 activity, while resveratrol treatment for reloaded muscles improved the cross-sectional area of fast-twitch muscle fibers, sirtuin-1 content and progenitor muscle cell count. In addition, curcumin and resveratrol treatment for unloaded muscles improved the satellite cell number. The authors encourage the use of curcumin and resveratrol due to their potential clinical effects against muscle disuse and atrophy to optimize the muscle regeneration process. 

Second, Gimenez-Donoso et al. [8] performed an outstanding experimental laboratory study which supported novel findings in a prevalent musculoskeletal condition such as myofascial pain syndrome, showing the first relationship in the research literature between this syndrome and nutritional biomarker status due to a fat rich diet producing acetylcholine spontaneous release in neuromuscular junction of mice. Spontaneous acetylcholine release at the neuromuscular junction was previously proposed as the key mechanism activating a vicious circle which perpetuates myofascial pain syndrome as a group of motor, sensitive and autonomic signs and symptoms originating from myofascial trigger points. This experimental laboratory study was carried out in male Swiss mice evaluating intramuscular adipocytes with Sudan-III and the plaque noise of the neuromuscular junction suggesting spontaneous acetylcholine release by electromyography. An increased plaque noise was presented after the interruption of the proposed diets. Thus, supplementation by a hypercaloric diet increased spontaneous neurotransmission in the neuromuscular junction promoting the development of myofascial trigger points.

Finally, Parsanathan et al. [9] performed an experimental laboratory study in high-fat-diet–fed mice with vitamin D deficiency, showing that vitamin D and L-cysteine cosupplementation mitigated biomarkers of musculoskeletal conditions. Vitamin D and L-cysteine cosupplementation provided beneficial effects for gene expression regarding myogenic biomarkers. Therefore, cosupplementation of vitamin D and L-cysteine improved the skeletal muscle biomarkers of musculoskeletal conditions more than monotherapy in vitamin D deficient high-fat-diet mice.

Future studies should address the role of ultrasound imaging as a promising tool for nutrient and biomarker status in musculoskeletal disorders [10]. The analysis of muscle tissue by sonoelastography may provide an alternative tool to indirectly evaluate sarcopenia and the lack of skeletal muscle mass from both qualitative and quantitative points of view, presenting a sensitivity of 77.3%, a specificity of 100% and a diagnostic accuracy of 87.5% [11]. The expression of mediators of neoangiogenesis and vascular density of the synovial characteristics in patients with rheumatoid arthritis provided a good correlation (*r* = 0.73) with the vascular area at histological level, linked to the cellular profile and pro-inflammatory cytokines, which provided considerable validity for using these measurements as objective assessments of synovial inflammation in clinical practice [12]. In addition, ultrasound analysis of the echo-texture, echo-intensity and echo-variation of the muscle tissue suggested promising results as biomarkers in neuromuscular pathologies such as amyotrophic lateral sclerosis [13]. Finally, the ultrasound software for the evaluation of muscle glycogen in the skeletal muscle may be decisive in detailing the musculoskeletal status and risk of injury in sport performance with promising results in this field, being a validated software with respect to muscle biopsy (*r* = 0.81) that allowed a non-invasive assessment of muscle glycogen [14,15].

In conclusion, the main findings of this Special Issue summarize the current scientific evidence available about nutrients and biomarkers in musculoskeletal diseases related to the metabolic impairment secondary to COVID-19 confinement, the prevention of osteoporosis for patients suffering from inflammatory bowel diseases, and nutritional issues in patients who suffer from spinal muscular atrophy, as well as new insights from experimental animal models on pharmacological agents to enhance muscle tissue regeneration, spontaneous acetylcholine release in the neuromuscular junction of mice by hypercaloric diet supplementation increasing spontaneous neurotransmission and the consequent activation of myofascial trigger points to originate myofascial pain syndrome, and the improvement of myogenic biomarkers of musculoskeletal conditions and gene expression due to vitamin D and L-cysteine cosupplementation. Future studies should investigate the role of ultrasound imaging as a promising tool for nutrient and biomarker status in musculoskeletal disorders.

## Figures and Tables

**Table 1:** The characteristics of the review studies included in the Special Issue about nutrients and biomarkers in musculoskeletal diseases.

Month, Year and Authors	Study Design and Characteristics	Main Findings
May, 2020. Martínez-Ferrán et al. [4]	Narrative review including 66 references.	Metabolic impairment consequences of COVID-19 physical inactivity, overweight, sedentary lifestyle, overfeeding and dietary intake modifications may predispose to musculoskeletal disorders. The restriction of calorie intake was proposed to prevent the COVID-19 confinement impact on physical inactivity and musculoskeletal conditions.
June, 2020. Ratajczak et al. [5]	Narrative review including 134 references.	Protein, fat, carbohydrate, vitamin, mineral, microelement and polyphenol intakes may predispose to osteoporosis and reduced bone mineral density in patients suffering from inflammatory bowel diseases. The development of nutritional guidelines to prevent osteoporosis for patients suffering from inflammatory bowel diseases should especially take into account vitamin D and calcium for bones mineral density, as well as vitamins such as A, B_12_, C and K, calcium, folic acid, magnesium, phosphorus, zinc, sodium, selenium and copper for bones mass formation.
December, 2020. Li et al. [6]	Narrative review including 85 references.	Lipid metabolic alterations, glucose metabolic modifications and vitamin levels alteration may be linked to neurodegenerative diseases such as spinal muscular atrophy. Dietary issues monitoring by biomarkers and nutrients may determine nutritional status and therapeutic target in patients who suffer from spinal muscular atrophy.

**Table 2:** Characteristics of the original research reports included in the Special Issue about nutrients and biomarkers in musculoskeletal diseases.

Month, Year and Authors	Study Design and Characteristics	Main Findings
June, 2020. Mañas-García et al. [7]	Experimental laboratory study in female mice (10 weeks old, weight ~20 g).	Pharmacological agents increased sirtuin-1 activity related to curcumin and resveratrol enhanced muscle tissue regeneration. Authors claimed potential clinical effects of these phenolic compounds for muscle disuse and atrophy to improve muscle regeneration.
October, 2020. Gimenez-Donoso et al. [8]	Experimental laboratory study in young (45–50 days) adult Swiss male mice.	A fat rich diet produced acetylcholine spontaneous release in neuromuscular junction of mice. Authors claimed that hypercaloric diet supplementation increased spontaneous neurotransmission in the neuromuscular junction suggesting the consequent activation of myofascial trigger points to originate myofascial pain syndrome.
November, 2020. Parsanathan et al. [9]	Experimental laboratory study in male mice (5 weeks old, 20–24 g).	Vitamin D and L-cysteine cosupplementation produced an improvement of myogenic biomarkers of musculoskeletal conditions and gene expression. Authors revealed that this cosupplementation improved muscle biomarkers linked to musculoskeletal conditions more than monotherapy.

## Data Availability

Not applicable.
